# Neuromuscular Ultrasound in Intensive Care Unit-Acquired Weakness: Current State and Future Directions

**DOI:** 10.3390/medicina59050844

**Published:** 2023-04-27

**Authors:** Felix Klawitter, Uwe Walter, Hubertus Axer, Robert Patejdl, Johannes Ehler

**Affiliations:** 1 Department of Anesthesiology, Intensive Care Medicine and Pain Therapy, Rostock University Medical Center, Schillingallee 35, 18057 Rostock, Germany; 2 Department of Neurology, Rostock University Medical Center, Gehlsheimer Straße 20, 18147 Rostock, Germany; uwe.walter@med.uni-rostock.de; 3 Department of Neurology, Jena University Hospital, Am Klinikum 1, 07747 Jena, Germany; hubertus.axer@med.uni-jena.de; 4 Department of Medicine, Health and Medical University Erfurt, 99089 Erfurt, Germany; 5 Department of Anesthesiology and Intensive Care Medicine, Jena University Hospital, Am Klinikum 1, 07747 Jena, Germany; johannes.ehler@med.uni-jena.de

**Keywords:** intensive care unit-acquired weakness, ultrasound, intensive care, critical illness myopathy, critical illness polyneuropathy

## Abstract

Intensive care unit-acquired weakness (ICUAW) is one of the most common causes of muscle atrophy and functional disability in critically ill intensive care patients. Clinical examination, manual muscle strength testing and monitoring are frequently hampered by sedation, delirium and cognitive impairment. Many different attempts have been made to evaluate alternative compliance-independent methods, such as muscle biopsies, nerve conduction studies, electromyography and serum biomarkers. However, they are invasive, time-consuming and often require special expertise to perform, making them vastly impractical for daily intensive care medicine. Ultrasound is a broadly accepted, non-invasive, bedside-accessible diagnostic tool and well established in various clinical applications. Hereby, neuromuscular ultrasound (NMUS), in particular, has been proven to be of significant diagnostic value in many different neuromuscular diseases. In ICUAW, NMUS has been shown to detect and monitor alterations of muscles and nerves, and might help to predict patient outcome. This narrative review is focused on the recent scientific literature investigating NMUS in ICUAW and highlights the current state and future opportunities of this promising diagnostic tool.

## 1. Introduction

Critically ill patients commonly suffer from extensive skeletal muscle atrophy and a generalized reduction in muscle strength, a condition termed intensive care unit-acquired weakness (ICUAW) [1]. Precisely, ICUAW is defined as a newly acquired weakness triggered by critical illness, immobilization and prolonged mechanical ventilation with a Medical Research Council Sum Score (MRC-SS) < 48 [2]. The presence of a flaccid, symmetrical paresis of the upper and lower extremities with sparing of the facial muscles and cranial nerves is considered a hallmark symptom in ICUAW [3]. Hereby, other neuromuscular complications with similar clinical symptoms (e.g., entrapment neuropathies due to prolonged bed rest and steroid-induced myopathies) have to be excluded. In ICUAW, the long-term outcome can be worsened by up to five years, even in milder cases with subtle levels of muscle weakness [4]. The pathophysiological correlate comprises the critical illness myopathy (CIM), the critical illness polyneuropathy (CIP) and the combined critical illness polyneuromyopathy (CIPNM) [5]. In daily clinical practice, ICUAW is predominantly diagnosed based on clinical examinations [6,7]. However, due to prolonged sedation, delirium and cognitive impairment, patient compliance is frequently disturbed and clinical assessment often limited [8]. Therefore, many compliance-independent diagnostic attempts including nerve conduction studies (NCS), electromyography (EMG), muscle biopsies, body fluid and in vivo biomarkers have been investigated to detect and monitor ICUAW [9,10,11,12]. Unfortunately, their applicability in daily clinical practice is currently limited [13]. To overcome the above-mentioned limitations, various medical imaging techniques have been evaluated in ICUAW and associated neuromuscular complications, with promising results. Magnetic resonance imaging (MRI) of skeletal muscles demonstrates distinctive alterations in the involved muscles, including hyperintensity in T2-weighted and short-tau inversion recovery (STIR) sequences [14,15,16]. These signal patterns are suggestive of edema as the underlying pathomorphological substrate and have been demonstrated to resolve in parallel to clinical recovery of several months [17,18]. Persistent MRI alterations correlate with prolonged weakness and disability [19]. In a single case, myosin loss has been proven by histopathology in a biopsy from muscle with the aforementioned imaging abnormalities [20]. Muscle MRI, and STIR sequences, in particular, may be useful in discriminating patients with critical illness myopathy from patients with immune mediated neuropathy [21]. Despite a slowly-growing interest in MRI as a potential diagnostic tool for patients at risk of ICUAW, the number of published cases is still very limited and the effort to conduct MRI studies is remarkable in the affected patient population. In contrast, ultrasound has been proven to be a valuable diagnostic tool for many different diagnostic approaches in intensive care medicine [22,23]. Especially for the assessment of pathologies of muscles and nerves, neuromuscular ultrasound (NMUS) is an established complementary method [24]. NMUS offers several advantages such as widespread availability, real-time assessment and non-invasiveness, and it can be used even in non-compliant patients [25]. In the last few years, extensive research has been conducted to investigate the value of NMUS in ICUAW and associated neuromuscular complications [26,27,28]. Hereby, NMUS might be of particular value for the detection, monitoring and, at least in part, outcome prediction in cases of ICUAW [29,30]. Beyond ultrasound, subsequent computational image analysis has also emerged in the last decade and might offer further improvement in diagnostic performance in ultrasonographic assessment [31,32]. Therefore, this narrative review aims to highlight the current scientific literature about the potential use of NMUS to diagnose and monitor ICUAW. Furthermore, recent and future developments in the field of computational image analysis will be discussed, focusing on their value for ultrasonographic applications in neuromuscular disorders within the ICU.

## 2. Basics of NMUS in the Intensive Care Setting

### 2.1. Basics for the Examination

**Figure 1:**
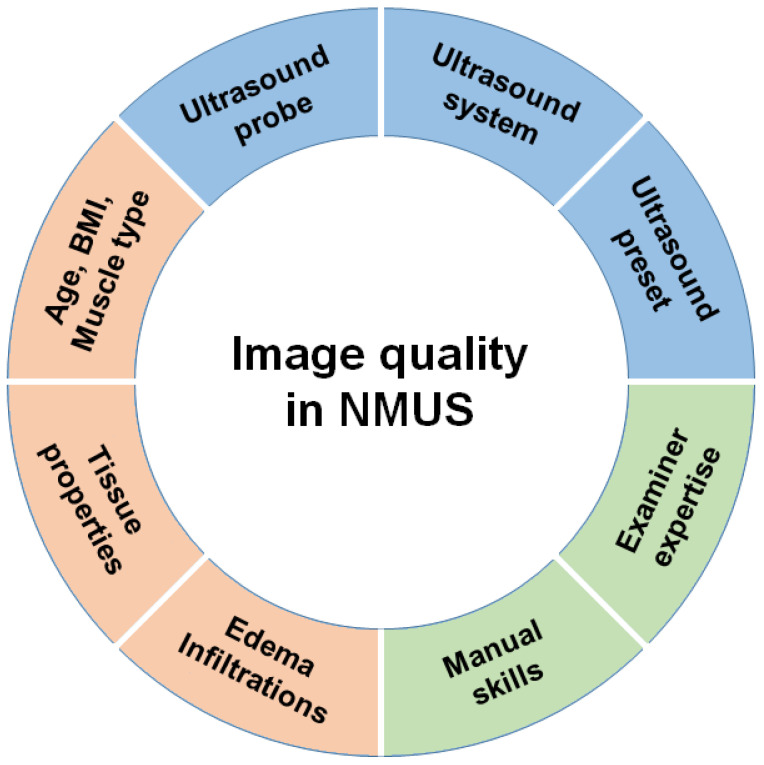
Factors contributing to image quality in NMUS. Blue sections: technical factors regarding the hardware of the ultrasound machine. Green sections: factors regarding the ultrasound examiner. Red sections: factors regarding patient and tissue characteristics.

Ultrasound can be used for a great variety of different assessments in intensive care medicine, but the underlying biophysical principles for ultrasonographic image generation remain the same [33]. Depending on the individual properties of tissues, mechanical soundwaves vary in their propagation speed, and the direction and intensity of these soundwaves can be modulated at tissue borders, leading to reflection, scattering or absorption. The fundamental principle behind the technical utilization of ultrasonography comprises the so-called piezoelectric effect, describing the interchanging transduction of soundwaves in electrical signals and vice versa. Macroscopic pathologies can be depicted by ultrasound because the biophysical properties of diseased tissues change in specific patterns. The great variation of hardware applications builds one important factor among the many which have to be considered in ultrasound imaging (Figure 1). In NMUS, different types of ultrasound probes can be used, depending on the targeted structure [34]. Hereby, the resolution and penetration depth are inversely proportional to each other as the frequency increases. Convex ultrasound probes often operate in the frequency range of 3–5 MHz and are suitable for the imaging of deeper structures. They also capture a larger image section than other ultrasound probes and are, therefore, suitable for depicting a larger region of interest, e.g., big muscles such as the quadriceps femoris (QF). However, for NMUS, linear transducers with a working frequency between 10 and 20 MHz are most commonly used [35]. For the assessment of small muscles and nerves, so-called “hockey stick” ultrasound probes can be used, providing high resolution imaging of superficial structures within a compact area [36].

Besides the selection of the appropriate ultrasound probe, attention should also be paid to optimizing the settings of the ultrasound system. The imaging depth should be adjusted in regard to the size of the targeted structure, and the focus position should be placed at the same level as the region of interest. Gain and power can be regulated to provide optimal visibility and image resolution. Predefined manufacturer setups, with specifically customized configurations of these parameters dependent on the targeted structure, are often available for a great variety of applications and can help to optimize ultrasound imaging quality. One example is the musculoskeletal setup, which can be used for the imaging of muscles, joints and nerves [24]. 

Furthermore, a proper handling of the ultrasound machine as well as the ultrasound probes is mandatory for good image acquisition. Hardware components should correspond to the currently examined structures and require permanent adjustment of probe positioning, minimization of external pressure applied by the probe and straight perpendicular alignment with reference to the underlying tissues. Basic skills in NMUS can be acquired through a diverse range of practical courses. Hereby, it has been shown that only minimal preparation is necessary to achieve good levels of reliability and validity in NMUS [37,38,39].

### 2.2. Specialties in the Intensive Care Setting

Critically ill patients are frequently sedated and mechanically ventilated and, therefore, are usually not compliant to participating actively in the ultrasound examination. Based on our own observations, access to the patient or the targeted structures can be limited by extensive wound bandages, intravenous catheters, extracorporeal membrane oxygenation (ECMO) cannulas or prone positioning. Therefore, NMUS in the ICU can be challenging and the operator must try as best as they can to establish optimal conditions for the examination. A supine position is favourable for most ultrasound assessments and, if possible, the extremities should be placed freely aside in slight flection without tension. Care must be taken to avoid unintended dislocation of monitoring devices, catheters and drainages. After adequate positioning, the optimal placement of the ultrasound probe has to be determined. This can be done, for example, based on topographical features such as bony prominences. In this context, the scientific literature contains a large number of ultrasound protocols with great variability in the methodology [40]. However, recent evidence suggests that different ultrasonographic approaches are equally effective in detecting muscular alterations in critically ill patients [41]. Additionally, patient-specific factors can influence the quality of the NMUS examination as well. Age, subcutaneous tissue thickness, muscle type, as well as an increase in connective tissue, fiber necrosis and inflammatory infiltrates, can impair the identification of muscles and nerves, thereby lowering the reliability and validity of NMUS results [24,42,43].

## 3. Current State of NMUS in ICUAW

### 3.1. Skeletal Muscles

Skeletal muscles vary in their form, size and architectural structure within and between individuals. Therefore, it is not surprising that sonographic images of skeletal muscles can also have some individual characteristics. In healthy subjects, the general ultrasonographic depiction of skeletal muscles appears black with a variable proportion of echo-rich connective tissue structures within. This picture is often called a “starry night” appearance [25]. In the absence of connective tissue, muscle fibers reflect lesser soundwaves back to the ultrasound probe, resulting in a relatively dark ultrasound image with a low echogenicity. It must be noted that healthy skeletal muscles can vary in their echogenicity, even without a pathological reason [29,32]. Most frequently, limb skeletal muscles have been assessed with NMUS in regard to investigating ICUAW. All studies listed in Table 1 are single-center trials, most of them with a prospective design. The study cohorts are heterogeneous with relatively small patient numbers ranging from 17 to 95. In particular, the QF and the tibialis anterior muscle (TA) have been examined frequently [26,27,28,29,30,44,45]. Besides limb skeletal muscles, the diaphragm has also been targeted by numerous workgroups and will be discussed later in this article [46,47,48]. Only a few trials assessed intercostal, abdominal or deep back muscles with ultrasound.

### 3.2. Peripheral Nerves

The nerve ultrasound has become an established complementary diagnostic approach in many different disease entities and syndromes, including peripheral nerve injuries, entrapment neuropathies and (poly)neuropathies [24]. At the ICU, sonographic assessment of the optic nerve sheath diameter can help to non-invasively detect increased intracranial pressure [50]. However, in regard to ICUAW, data on the diagnostic value of peripheral nerve ultrasound are limited (Table 2). Most of the corresponding studies are single-center prospective clinical trials with a mixed patient population. Nerves of the upper and lower extremities have been assessed describing changes in nerve thickness, nerve CSA and nerve echogenicity. In one study intraneural vascularization was also quantified [45]. 

**Figure 2:**
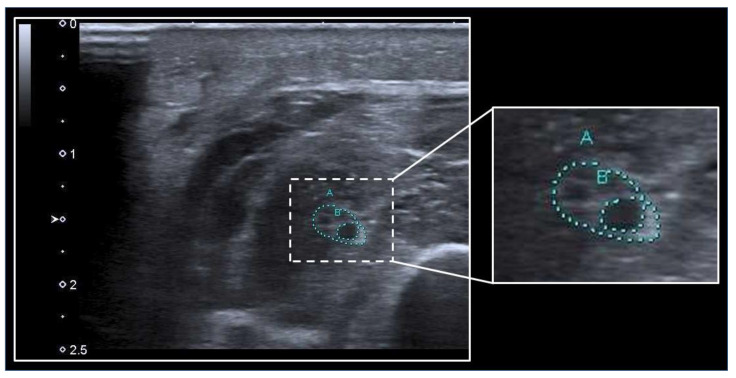
Assessment of nerve CSA with ultrasound. The median nerve (**A**) at the mid to proximal forearm is surrounded by muscles and can be identified by the heterogeneous “honeycomb” pattern within the echorich perineurium. A swollen fascicle (**B**) within the nerve depicts as a hypoechogenic area. The nerve CSA can be calculated using the tracing function of the ultrasound machine.

The nerve CSA is one of the most frequently assessed neural parameters in regard to ICUAW. Hereby, the peripheral nerve can be depicted in sonographic short axis and its circumference can be manually traced (Figure 2). However, data on measurements of the nerve CSA in ICUAW are sparse and partly inconsistent, whereby nerve CSA does not seem useful in distinguishing between patients with and without ICUAW but may be of value in distinguishing patients with a predominant presentation of CIP from patients with CIM [44,45,51,52]. In general, an increased nerve CSA seems to correlate with the duration of mechanical ventilation and days spent at the ICU [52].

Nerve echogenicity is another parameter that can be assessed using NMUS. Hereby, the overall brightness of the peripheral nerve is obtained. Similarly to with the nerve CSA, evidence is sparse in regard to ICUAW and associated subtypes. In CIP, a significant decrease in nerve echogenicity has been described [45], which is contrary to findings from patients with chronic inflammatory demyelinating polyneuropathies, where the disease severity correlates with a higher nerve echogenicity [54]. However, the echogenicity of peripheral nerves seems unsuitable in distinguishing CIP from other polyneuropathies [53].

### 3.3. Diaphragm

The diaphragm is considered the most important striated skeletal muscle in maintaining ventilation [55]. Neuromuscular diseases affecting diaphragmatic muscle activity can, therefore, significantly alter both respiratory activity and gas exchange. Critically ill patients are frequently affected by paresis of the diaphragm, as even short-term mechanical ventilation is considered a key risk factor for ventilator-induced diaphragmatic dysfunction (VIDD) through the induction of muscle fiber atrophy [56]. In addition, lower diaphragmatic muscle mass has been shown to be associated with an increased risk of weaning failure and worsened outcome [57]. In recent years, sonography of the diaphragm has been intensively investigated as a possible diagnostic method for VIDD and is now considered a valid, easily reproducible tool with prognostic significance [50]. In particular, diaphragmatic ultrasound has been used to investigate both static (diaphragmatic thickness) and dynamic (thickening fraction, maximum diaphragmatic excursion) parameters [47,48]. Furthermore, diaphragm echogenicity and tissue Doppler imaging have recently also been proposed as possible surrogates for diaphragm function [58,59]. Although VIDD and ICUAW are common in critically ill ICU patients, the interplay between these two entities remains unclear [60,61]. Irrespective of this, the simultaneous presence of ICUAW and VIDD is associated with a higher risk of weaning failure, compared to the presence of one of these conditions alone [62,63]. However, the value of diaphragmatic sonography as a surrogate parameter for generalised limb muscular weakness in ICUAW seems questionable. Jung et al. were able to demonstrate concomitant VIDD in 80% of a cohort of 40 ICUAW patients, but without correlation between diaphragmatic ultrasound parameters and limb muscle strength [64]. This is in line with results by Vivier and co-workers, who were also unable to show a significant correlation between the parameters of diaphragmatic dysfunction and limb muscle strength [65]. In a recent study, a decrease in diaphragmatic muscle thickness was observed in ICU patients, but without a significant difference between patients with and without ICUAW [26].

### 3.4. Parameters of Muscle Quantity and Their Diagnostic Value for ICUAW

Critically ill patients frequently suffer from an early and progressive reduction in muscle mass. A recent meta-analysis suggests that a daily loss of about 2% can be estimated, whereby ultrasound is the most frequent used method to detect and monitor muscle degradation [66]. Hereby, surrogate parameters for the quantification of muscle mass are the muscle layer thickness (MLT) and the muscle CSA [67]. The MLT is usually determined in sonographic short axis as the maximum vertical diameter of muscle tissue within the muscle fascia. The MLT has been validated in clinical trials and anatomical studies with excellent intra- and interrater reliability [68,69,70,71]. However, in critically ill patients, estimation of muscle mass by a single MLT measure might be insufficient [72]. In contrast, the MLT seems suitable for the repetitive assessment and monitoring of muscle wasting within the ICU stay [73,74,75,76]. Recent evidence suggests that the loss of muscle mass is more severe in critically ill patients with ICUAW [30,73]. For the prediction of patient outcome, MLT has been shown to predict prolonged intensive care treatment and functional outcome [76,77]. Furthermore, a reduction in MLT is associated with an increased mortality in ICUAW patients [30].

Similarly to with the nerve CSA, the skeletal muscle CSA can be calculated to estimate muscle mass. Hereby, the anatomical rather than the physiological muscle CSA is measured [33]. Analogous to the MLT, in NMUS the muscle CSA is measured in the sonographic short axis view of superficial limb skeletal muscles such as the biceps brachii muscle (BB), QF and TA [12,73,74,75]. Some muscles such as the QF cannot be depicted fully in one image frame, so the individual muscle components (in the case of the QF: rectus femoris muscle, vastus intermedius muscle, vastus lateralis and vastus medialis muscles) have to be evaluated separately. Similarly to with the MLT, a decrease in muscle CSA of up to 30% has been demonstrated in ICU patients after several days of critical illness [12,74,76], whereby patients with ICUAW seem to have greater losses of muscle mass compared to patients without ICUAW [73,78]. The morphological and molecular equivalents of these sonographic observations include muscle fiber atrophy, necrosis and muscle protein breakdown [12,79]. Furthermore, sonographic assessment of the muscle CSA can help to predict patient outcome. A reduction in muscle CSA has been shown to correlate with a significant reduction in muscle force and function [74,76]. Overall, a reduction in muscle mass during critical illness seems to be associated with increased mortality in patients with ICUAW [30,78].

### 3.5. Parameters of Muscle Quality and Their Diagnostic Value for ICUAW

The ultrasonographic brightness of a muscle can be described by the muscle echogenicity (ME). Muscle tissue from healthy subjects is usually relatively dark in appearance with a variable degree of interspersed bright echo signals from intramuscular connective tissue. Hereby, most of the ultrasound waves pass through the muscle tissue without being reflected. The brighter the muscle appears the more ultrasound waves are reflected back to the transducer. However, neuromuscular disorders can alter the ME in many different ways, including heterogeneous patterns of hyper- and hypoechogenicities [25]. In contrast, ICUAW and associated neuromuscular disorders seem to increase the brightness of the entire muscle during critical illness [28]. 

Most studies investigating ME in critically ill patients use the so-called greyscale analysis. A computer-based assessment of digitally stored ultrasound images allows quantification of image brightness by calculating the greyscale values of pixels within a defined region of interest. This method is, mostly, not implemented in current ultrasound machines, so quantification of ME using greyscale analysis requires a semi-automated secondary assessment by the examiner. Greyscale analysis has been validated in non-ICU and ICU patients with neuromuscular disorders [32,80], whereby the accuracy depends on the size of the measured image area [81]. Furthermore, software-based greyscale analysis has also been investigated in patients with ICUAW [29,45,49]. Hereby, this method seems unsuitable in detecting ICUAW, and the correlation with clinical outcome data was low [29,45]. In contrast, Naoi et al. reported a specificity of about 75% and a sensitivity of 69.2% for upper arm ME in detecting ICUAW, but without further specification of significant differences in ME between patients with and without ICUAW [49]. 

**Figure 3:**
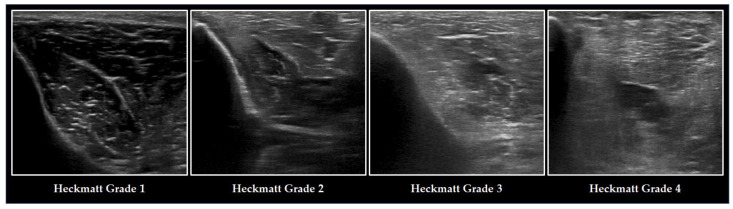
Ultrasonographic changes of the TA and the adjacent tibia in ICUAW graded with the Heckmatt Scale. The image on the left refers to a healthy individual, graded with lowest Heckmatt grade 1. Muscle tissue is classically dark with some bright echo signals from connective tissue. In critical illness (from the second left image) and especially in patients with ICUAW (from the second right image), muscle tissue becomes more echointense (Heckmatt grade 2) and the bone echo begins to become blurry (Heckmatt grade 3), until it has nearly vanished (Heckmatt grade 4).

Another method to assess ME is semi-quantitative grading using the Heckmatt Scale (Figure 3). Introduced by Heckmatt and coworkers in 1982, this four-point scale relies on the visual assessment of ME and bone echotexture [82]. The brighter the muscle and the lower the bone echogenicity, the higher the grading. The Heckmatt Scale demonstrates good reliability [32] and has been evaluated in critically ill patients [28]. Furthermore, it has been demonstrated that the Heckmatt Scale can be a useful tool to identify patients with ICUAW [10,27,29]. Recent evidence suggests that the Heckmatt Scale might be superior to software-based greyscale analysis for the assessment of ME in ICUAW [29], but inherent limitations of this visual approach must always be kept in mind [31]. 

Furthermore, assessment of ME can help to predict patient outcome. Several studies demonstrated that an increase in ME was correlated with the severity of muscle weakness and overall impairments in functional patient outcome [29,49,74,76]. The presence of increased ME has also been previously associated with increased mortality in patients with CIPNM [27].

## 4. Future Directions

Over the last two decades, ultrasound has been introduced more and more into the evaluation of various neuromuscular disorders in different patient populations. Expert opinion and interpretation set up the basis for diagnosing and characterising pathological findings, and for distinguishing normal and abnormal depiction of muscles and nerves using NMUS. However, the operator-dependent assessment might be biased by several factors, including examiner experience, technical impairments, optical illusions and patient characteristics, interfering with the diagnostic value of ultrasound assessments. Computer-based semi-automated image analysis was introduced to support ultrasound image interpretation and to add a more objective approach to image quantification in neuromuscular disorders [31,32]. However, segmentation and measurement require manual operations, making complex image analysis unattractive for a broad application in terms of effectiveness. In this context, artificial intelligence (AI) may offer to be a promising tool in implementing image analysis in research and daily clinical practice. Hereby, AI refers to operator-independent computer learning, abstraction and problem solving [83]. In recent years, AI has been implemented in many different medical disciplines in order to deal with the exponentially growing amount of data. Especially in the field of medicine, big imaging data from various modalities crucially depend on effective and reliable methods to correctly assess and interpret their diagnostic results [84]. Regarding modes of operation, AI can be roughly divided into machine learning (ML) and deep learning (DL) [85]. Whereas ML depends on predefined features enabling the computer to identify certain characteristics and structures according to the manual input, DL takes it a step further by independently extending predefined characteristics based on repeated example processing [84]. This enables DL architectures to solve more complex problems and recognize abstract patterns, already exceeding human performance in some cases. 

In regard to NMUS, the application of DL has already been investigated with promising results. The parameters of muscle quantity (MLT and muscle CSA) and quality (ME and PA) in healthy and pathological conditions have been tested, with good to excellent accuracy compared to manual assessment by trained operators [86,87,88,89,90]. As increased ME can hamper a clear identification of muscle within surrounding tissue, the accuracy of DL in myopathic conditions with pathological elevated ME seems questionable. However, a study by Marzola and coworkers shows that, even in conditions of increased ME, the automated calculation of quantitative parameters seems possible with good diagnostic performance [91]. Furthermore, recent investigations demonstrate that DL seems to be superior in identifying pathological patterns and myositic alterations within muscle tissue in NMUS [92,93]. In another study, DL was used to classify the physical function of patients with Duchenne muscular dystrophy (DMD) based on the results of a NMUS assessment [94]. The cohort consisted of 85 patients with confirmed DMD, exhibiting different clinical stages of disease severity, ranging from normal muscle function to marked impairments of the upper and lower extremities. DL achieved excellent performance in differentiating ambulatory and non-ambulatory patients by analysing their NMUS images, underlining the potential of AI-based applications to further improve diagnostic performance of NMUS in the huge field of neuromuscular disorders.

Unfortunately, up to the present date, no studies have evaluated the value of automated image analysing technologies in ICUAW and associated neuromuscular complications, but this might be a promising attempt in future studies. Besides the advantage of possibly a more comprehensive image analysis, AI could also help to optimize current NMUS protocols and ultrasound parameter adjustments, further promoting the implementation of NMUS and post-ultrasound image analysis into the daily clinical practice of intensive care medicine. 

## 5. Conclusions

In conclusion, NMUS is a promising, non-invasive alternative diagnostic approach for the assessment of ICUAW in critically ill patients. This could be of value, especially in non-compliant and neurologically impaired patients, where a classical assessment of ICUAW is hampered. Furthermore, NMUS might also be helpful in the prediction of patient outcome. The development of consistent ultrasound protocols and validation in accurately powered patient cohorts is needed to establish NMUS as a widespread bedside- available diagnostic tool in intensive care medicine. Beyond basic ultrasound assessment, the implementation of automated image analysis might further improve diagnostic performance and objectivity in NMUS. 

## Figures and Tables

**Table 1:** Non-systematic selection of studies investigating skeletal muscles with NMUS in ICUAW within the last 10 years. BB: biceps brachii muscle. BRA: brachialis muscle. BR: brachioradialis muscle. CB: coracobrachialis muscle. CSA: cross-sectional area. EDL: extensor digitorum longus muscle. FCR: flexor carpi radialis muscle. FE: forearm extensor muscles. FIM: Functional Independence Measure. IC: intercostal muscles. ICUAW: intensive care unit-acquired weakness. LOS: length of stay. ME: muscle echogenicity. MMT: Manual Muscle Testing. MRC-SS: Medical Research Council Sum Score. mRS: modified Rankin Scale. PA: pennation angle. QF: quadriceps femoris muscle. RF: rectus femoris muscle. TA: tibialis anterior muscle.

Author	Year of Publication	Study Design	Patients (n)	Study Cohort	Assessed Muscles	Study Endpoints
Naoi et al. [49]	2022	retrospective, single-center	34	non-surgical	BB, BRA, TA, EDL	Relations between ME, MRC-SS, MMT and FIM
Klawitter et al. [29]	2022	prospective single-center	51, 38 with image analysis	perioperative	BB, BR, QF, TA	Differences in ME between ICUAW and non-ICUAW patients; correlation of ME with MRC-SS, mRS, Barthel Index
Paolo et al. [26]	2022	prospective single-center	50	medical, surgical	RF, IC	Changes in RF-CSA, PA, diaphragm and intercostal muscle thickness
Patejdl et al. [10]	2019	prospective single-center	18	perioperative	BB, BR, QF, TA	Changes in ME; serum biomarker; correlation with mRS
Kelmenson et al. [27]	2018	prospective single-center	95	medical, cardiac, (neuro)surgical	upper arm and upper thigh muscles	Diagnostic accuracy of single nerve conduction studies and NMUS
Hadda et al. [30]	2018	prospective single-center	70	non-surgical	BB, CB, QF	Changes in muscle thickness up to 90 days; LOS in hospital, mechanical ventilation time, survival rate
Witteveen et al. [45]	2017	prospective single-center	71	medical, surgical	BB, TA, RF, FCR	Muscle thickness; muscle echo intensity; median nerve CSA
Grimm et al. [28]	2013	prospective single-center	28	neurological	BB, FE, QF, TA	ME; number of muscle fasciculations

**Table 2:** Non-systematic selection of studies investigating peripheral nerves with NMUS in ICUAW. CSA: cross-sectional area. ME: muscle echogenicity.

Author	Year of Publication	Study Design	Patients (n)	Study Cohort	Assessed Nerves	Study Endpoints
Gruber et al. [51]	2022	prospective, single-center	16	neurological	median, ulnar, fibular	nerve CSA; ultrasound pattern sum score
Bulinski et al. [52]	2022	prospective, single-center	17	neurological, neurosurgical	peroneal, tibial, sural	nerve CSA
Erdmann et al. [53]	2022	retrospective, single-center	66	mixed neurological	median, ulnar, radial, fibular, tibial	nerve echogenicity
Fisse et al. [44]	2021	prospective, single-center	35	medical, neurological	median, ulnar, radial, tibial, sural, vagal	nerve CSA; ME; cytokine analysis
Witteveen et al. [45]	2017	prospective, single-center	71	medical, surgical	median, peroneal	muscle thickness; ME; nerve CSA; nerve thickness; intraneural vascularization

## Data Availability

Not applicable.
